# HCV treatment access among Latinxs who inject drugs: qualitative findings from Boston, Massachusetts, 2016

**DOI:** 10.1186/s12954-019-0314-6

**Published:** 2019-07-09

**Authors:** Avni Mittal, Karen C. Kosinski, Thomas J. Stopka

**Affiliations:** 1 Department of Public Health and Community Medicine, Tufts University School of Medicine, 136 Harrison Avenue, Boston, MA 02111 USA; 2 Department of Community Health, Tufts University School of Arts and Sciences, 574 Boston Avenue, Suite 208, Medford, MA 02155 USA; 3 Department of Public Health and Community Medicine, Clinical and Translational Sciences Institute, Tufts University School of Medicine, 136 Harrison Avenue, Boston, MA 02111 USA

**Keywords:** Hepatitis C virus, Latinxs, Boston, Direct-acting antivirals, Qualitative research, People who inject drugs

## Abstract

**Background:**

Compared with Caucasians, Latinxs with the hepatitis C virus (HCV) tend to initiate treatment less often, discontinue treatment, become infected younger, and have higher reinfection rates post-treatment. Little is known about HCV treatment experiences among Latinxs who inject drugs in the Northeastern USA.

We assessed knowledge, attitudes, and perceptions tied to HCV, as well as HCV treatment readiness, and explored the overall HCV treatment experience of Latinx people who inject drugs (PWID) in Boston.

**Methods:**

We conducted qualitative interviews with monolingual and bilingual Spanish-speaking Latinx PWID (*n* = 15) in Boston, Massachusetts, between 2015 and 2016. We used a thematic content analysis approach to code and analyze data to identify knowledge, attitudes, and experiences related to HCV treatment.

**Results:**

We identified barriers and facilitators to HCV treatment. Six salient themes emerged from the data. For participants who had not initiated HCV treatment, lack of referral, fear of quitting drugs, and fear of relapse were perceived barriers. Trust in medical providers and a willingness to quit drugs were primary facilitators. Most participants had positive HCV treatment experiences, and several emphasized the need for outreach to Latinxs about the advantages of newer treatment options. Concerns about HCV reinfection were also notable.

**Conclusions:**

We identified a range of experiences tied to HCV treatment among Latinx PWID. HCV care providers play a key role in determining treatment uptake, and more treatment information should be disseminated to Latinx PWID. Healthcare providers should capitalize on treatment facilitators by ensuring referrals to treatment and should continue to address perceived barriers.

## Background

The hepatitis C virus (HCV) is a blood-borne virus [1] that is mainly transmitted through unsafe injection drug use [2]. In the USA, between 2.7 and 3.9 million people are living with chronic HCV [3], and from 2010 to 2015, HCV incidence increased by 294% with the highest rates among young people who inject drugs (PWID) [4]. Among infectious diseases in the USA, HCV is the leading cause of death killing 19,659 people in 2014 [3]. HCV deaths have risen in the past 15 years and currently surpass total deaths in the USA attributable to 60 other nationally notifiable infectious conditions combined [5]. Latinxs are the largest minority group in the USA and, as of 2015, comprised 17.6% of the population [6]. Compared with Caucasian counterparts, Latinxs living with HCV tend to initiate treatment less often, discontinue treatment more often, become infected with HCV at an earlier age, and have higher re-infection rates following treatment [7]. HCV incidence rates among Latinxs have been increasing; currently, 4% of the Latinx population in the USA has HCV [8].

In 2015, 9079 cases of HCV were reported in Massachusetts [9], giving Massachusetts the highest reported infection rate in the country at 3.7 per 100,000 population (national rate 0.8 per 100,000) [4]. Between 2002 and 2009, there was a 78% increase in HCV cases among 15–24 year olds in Massachusetts, and between 2002 and 2013, reported HCV cases increased by 137% among 15–29 year olds [2, 10]. Seventy-two percent of HCV cases were associated with current or past injection drug use [2]. In 2015, 14% of HCV-infected individuals in Boston were Latinx [9], but little is known about the context that surrounds infection rates and HCV treatment-seeking behaviors.

Starting in 2013, direct acting antiviral (DAA) therapies, which have demonstrated high treatment success rates, few side effects, and short treatment regimens (8–12 weeks), began to offer new hope that public health officials and clinical experts could begin to curb the HCV pandemic. Despite progress, many barriers to HCV treatment have hindered success, such as low access to DAAs [11], especially for PWID [12], sobriety requirements prior to treatment access [11, 13], and lack of treatment for people without advanced liver disease [11–14]. Medicaid and private insurance sobriety and advanced liver disease requirements vary by state, from no restrictions in Massachusetts and Connecticut, to 1 year of sobriety and F3 or F4 fibrosis level requirements in Louisiana in 2017 [13]. Additionally, after controlling for sociodemographic, clinical, and system factors (e.g., age, sex, insurance status, severity of fibrosis stage, and other comorbidities), compared to non-Latinx whites, Latinxs were significantly less likely to receive HCV treatment, even in the new DAA era [15].

Previous studies have looked at perceptions of HCV treatment and treatment readiness among Latinx PWID, but none were conducted with Spanish-speaking individuals [16, 17]. In Boston, there is little community-based research focused on Latinx PWID, and even less is known about HCV treatment perceptions among Spanish-speaking PWID, who tend to be more recent arrivals to the USA. We conducted a qualitative study with Spanish-speaking PWID in Boston to assess knowledge, attitudes, and experiences tied to HCV, as well as HCV treatment readiness, and explore the overall HCV treatment experience of Latinx PWID in Boston.

## Methods

### Study sample

We recruited a purposive sample of Latinx PWID through street outreach, outreach in local community-based organizations, and clinical referrals. This study was part of a larger study of HCV treatment readiness (Tufts Responds to the Epidemics of Addiction and hepatitis C Together [Tufts REACTs]; PI: Stopka). Participants were eligible if they were residents of Boston, 18–55 years of age, monolingual (Spanish) or bilingual (Spanish-English), reported current or previous illicit drug injection histories, and reported to be either living with HCV or had recently received HCV treatment. We define an illicit injected drug as any injected drug that was not used as prescribed by a clinician or that was obtained illegally. HCV status was determined by self-report, but clinicians and program directors at social service institutions confirmed the participant’s HCV status for many participants when referring the person to our study. Of the 15 participants, seven were referred from local hospitals and five were referred from Latinx recovery support institutions after HCV status was confirmed. Three others were from street outreach or referral from Tufts REACTs, which is the parent study.

### Instrument

We developed, translated, and back-translated a semi-structured qualitative interview guide. Interview domains included drug injection history, social network composition, participant understanding of HCV and HCV treatment, experiences with healthcare providers, opinions on treatment readiness, facilitators/barriers to treatment, and cultural considerations related to drug use, relapse, and factors affecting treatment access.

### Data collection

Interviews (June to October 2016) were conducted by the lead author in a private interview room at the Tufts University School of Medicine or a local Latinx community-based agency and lasted approximately 60 min. Interviews were conducted in the participant’s preferred language (Spanish (*n* = 12), English (*n* = 2), or half/half = 2), were digitally recorded, and transcribed in the language of the interview by the lead author. Participants were compensated $25 for their participation.

### Coding and analysis

We used a thematic content analysis approach to develop analytical codes and themes; initial codes were based on interview questions and additional codes, which emanated from the data, and were added iteratively during the coding process (total 16 parent codes, 58 sub-codes/child codes). All coding and analyses were completed by the lead author, reviewed by the PI, and were conducted in a context analysis program (Dedoose v7.5.9, Boston, MA). We then reviewed coding reports to identify key themes in the data. Themes were considered salient if three or more participants shared the same sentiments or experiences. Themes among those who had not yet experienced treatment (*n* = 4) were considered salient when two participants shared similar experiences and perspectives. For improved readability without compromising content, we removed pauses, utterances, and colloquialisms from the quotes.

All participants provided verbal informed consent. This study was reviewed and approved by the Tufts University Health Sciences Institutional Review Board.

## Results

Study participants were predominantly men (87%; 13/15), Puerto Rican (93%; 14/15), and > 40 years of age (67%; 10/15) and had already received HCV treatment (73%; 11/15) (Table 1).

**Table 1:** Characteristics of Latinx PWID participants who participated in in-depth interviews, Boston, Massachusetts, 2016

Age	Gender	Ethnicity	Treated for HCV?
39	Man	Puerto Rican	Yes
28	Woman	Venezuelan and Guatemalan	No
56	Man	Puerto Rican	Yes
49	Man	Puerto Rican	Yes
33	Man	Puerto Rican	Yes
52	Transgender woman	Puerto Rican	Yes
50	Man	Puerto Rican	Yes
33	Man	Puerto Rican	No
49	Man	Puerto Rican	No
44	Man	Puerto Rican	Yes
48	Man	Puerto Rican	Yes
40	Man	Puerto Rican	Yes
47	Man	Puerto Rican	Yes
50	Man	Puerto Rican	Yes
44	Man	Puerto Rican	No

Six salient themes emerged from the data; we divided these six themes into two categories: (1) factors affecting HCV treatment readiness and uptake and (2) participant experience with HCV treatment. Findings tied to these categories (boldface text) and themes (in italics) are presented in detail below.

### Factors affecting HCV treatment readiness and uptake

#### Perceptions about HCV treatment access

Many participants experienced initial reservations about taking HCV treatment therapies. Participants were often deterred from HCV treatment because they knew that relatives and peers had experienced substantial side effects while taking older, interferon-based treatments. Participants who watched their peers suffer expressed initial fear to start treatment and feared experiencing similar side effects. One participant explained his initial fears about taking HCV treatment, even though he understood that the new treatment had different and less severe side effects:

Well my cousin, he also took the treatment but, the treatment that he took for hepatitis C, was with … injections … So I saw him very gaunt, and so I didn’t want, I didn’t dare to take it because I was a little fat, I had more to spare … so I was afraid to take it. But since the [new] treatment doesn’t have the same [side effects] … (taken treatment)

Despite initial fears about beginning treatment, participants almost unanimously (*n*=14/15) believed that HCV treatment was very easy to access and that one only had to ask for help to be put on treatment. They expressed that, as of 2016, people who remained untreated “were not trying hard enough.” Two participants exemplified these sentiments with the thoughts below.

You have to do it, you have to go to the hospital. You have to ask for help, because the doctor isn’t where you are, you have to go, ask for help, talk to the doctor, take the test, and tell the doctor that you truly want the treatment. That you want to be cured (taken treatment)

Although the following participant expressed similar sentiments, he had not yet obtained treatment, highlighting the remaining ambivalence among some PWID in the Latinx community.

It’s easy man. Nowadays it’s easy. We are in 2016. We are not in the 80’s. We are not in the 70’s. All you have to do is show up. Be accountable for your stuff you know what I’m saying? Show up and you’ll get treatment. Ask questions and you’ll get your answers. There’s nothing that hard. (Participant “A”—not taken treatment. See reasons in the “Barriers to treatment and treatment adherence” section)

Of the four participants who had not taken treatment, only one participant reported that he found it difficult to gain access to HCV care through his current primary care provider; he was still seeking a referral to an HCV specialist at the time of his interview.

Participants perceived Massachusetts and its healthcare staff to be a key destination for healthcare access and treatment. Participants indicated complete satisfaction with their experiences with Boston healthcare professionals and were grateful for the kindness and attention they received. Many specifically came to Boston for healthcare. The participant below relays his positive experience with the system:

Yah, everyone is so nice. Um Boston … I looked searched on the internet, and it’s a … site for the best hospitals I say. There is Brigham and Women’s … um, Mass General, BMC, Boston Medical Center. These hospitals that, I see people that were treated here. Here there is great security in the hospitals. It is like everyone, has problems, in all areas here. My experience here was fabulous. They gave me a kidney. I am healthy, I am being treated...the doctors are really amicable. (taken treatment)

Many participants described difficulties gaining access to HCV care in Puerto Rico and explained that Massachusetts was their best option.

I came out of jail, it was for one year in Puerto Rico. I saw that things were not equal. And well I decided, I said that the healthcare system in Puerto Rico was bad, and even though there are parts of the United States that are bad, it’s not like here. So I left for here, I left for Springfield, and like I told you, I started treatment. (taken treatment)

#### Barriers to treatment and treatment adherence

Although MassHealth (Medicaid in Massachusetts) covers more than one treatment course for HCV if reinfection occurs, physicians often find it difficult to advocate for patients who relapse when they appeal to insurance companies. Thus, patients may receive older DAA treatment plans that require more pills or have more side effects if they relapse [18]. However, it is possible to be approved for DAA treatment a second time. For several participants, the possibility of reinfection was seen as a barrier because physicians had explained that treatment is typically only given once, or that insurance covers treatment only once. These participants were therefore waiting until they were ready to stop drug use to begin treatment.

I got MassHealth and … let’s say like if I go right now to see my doctor and be like listen I wanna do the pill, they only pay for it one time because it’s so much money you know what I mean? And like I can’t do it a second time. Like if I do it a first time, and I succeed and I … don’t got it no more and I end up using again and I end up catching it again, then I’m gonna be with it all my life because they are not gonna pay for it again … It’s gonna be something that I have to really sit down and be like ok, I’m done with drugs." (Participant “A”, not taken treatment)

All participants reported that peers who have gone without treatment are too busy using drugs and thus were not thinking about HCV treatment. Two different participants described those peers as follows:

Those that are not interested because they don’t care about their life. They are still using drugs, they still drinking, they still doing this (taken treatment)

All of them are still on the street doing drugs... I saw this and said, not me. They don’t want to quit [using]. They want to continue using drugs. They are going to die. That is what I tell them. If they don’t try now, they are going to die. Either the drugs are going to kill them or the hepatitis will (taken treatment)

When participants were asked if people who were currently injecting drugs should receive HCV treatment, about half (*n* = 7) replied that they believed the treatment would not be effective if people were still using because there was a possibility of becoming re-infected after the person was cured. They felt that while using drugs, it was not possible to properly take the treatment and they did not think that people using would take it seriously. One participant explained, “If they are not ready to stop using drugs, they are just wasting their time, and other people’s time.” (not taken treatment)

When asked about barriers to treatment specific to Latinxs, many responded that they did not feel Latinxs were treated any differently than another race/ethnicity when trying to obtain treatment. “I believe that for Latino people, everyone is the same. For the treatment, in all the hospitals, for all the world, it is the same” (taken treatment). However, one participant believed Latinx were treated as “second class citizens” in the USA and added that the shame associated with being Latinx and living with addiction prevented many from seeking HCV care.

An American [that] has hepatitis [C] go to the hospital...and get cured. But for example, I have hepatitis C, I go to my doctor at Mass General, the best hospital in the world, and yet I haven’t asked for treatment. I don’t know why exactly but it has to do with shame … . Addiction is an embarrassment for a Hispanic family (not taken treatment)

The above participant had explained that she herself had delayed HCV treatment because she worried her liver was “too damaged” to be treated due to a family history of poor liver conditions. However, she also disclosed that she had an appointment with her PCP, whom she saw regularly for other health issues, the day of her interview to discuss treatment options as she “finally” felt ready for treatment.

Surprisingly, many primarily Spanish-speaking participants, and several with no English proficiency, reported few problems communicating with and understanding their providers. They reported that their providers spoke enough Spanish for them to understand or a translator was available. They reported the ability to understand side effects of treatment as well as the treatment plan from their providers without problems. However, participants noted that an in-person translator was not always available when needed. When participants were asked if they had any problems communicating with healthcare providers, one participant answered,

No because there always is a translator, and almost all, almost all of the doctors that I have had, by luck … have spoken a bit of Spanish or, understand. Sometimes there wasn’t a translator. Sometimes we speak like we are now. The little English I know and the little Spanish that he knows. (taken treatment)

#### Facilitators to HCV treatment readiness

Many participants were hesitant to start treatment or were concerned about experiencing difficulties during treatment. These participants reported that their physicians were invaluable in helping them become treatment-ready. Physicians helped quiet fears about side effects, explained how the virus worked, and discussed what participants could expect from the treatment.

Ok, when I found out what hepatitis C does to the liver … I talked to my doctor and she explained to me that I could lose the liver, the transfusions. So with the support of my doctor for the most part and it was my friends that told me, “Hey take [the medication], it is not bad”. So, I took it and already my life has changed. Now it doesn’t hurt here anymore, or over there. And at first I was afraid, afraid that something was going to happen to me, afraid of a reaction from the medication. But the doctor explained everything to me. (taken treatment)

Many participants, like the one below, described how they were motivated by their healthcare providers explaining that they were going to die without treatment. The hope of a new life and the fear of dying made participants more treatment ready. “Like my doctor told me. [The medication] does work. If you don’t want to die, then you have to take it” (taken treatment).

Participants cited several reasons for their interest in treatment. Many relayed an unwillingness to die as the primary reason. They hoped for a second chance at life and hoped to spend their remaining years with their loved ones and people with whom they had lost contact while using drugs. When the participant below was still seeking a referral to a specialist and was asked how he knew he was ready for treatment he answered,

I know people who have passed because their liver shuts down, or for whatever reason, that hep C takes a toll on your body. You can’t, filter things properly if your liver develops into the cirrhosis stage and stuff. I would like to get it not just for me, for my wife’s sake, my wife has hep C too … and I have two babies, I have a two-year-old and a four-year-old. And it’s like, at my age, do they deserve to have dad around? (not taken treatment)

When one participant who was not yet ready for treatment was asked how he would know when he would be ready, he explained that he needed to hit rock bottom and until then, he was not comfortable taking treatment. He expressed that once he was tired of his current lifestyle, he would consider taking the treatment. He believed he would need to be incarcerated for a period of time in order to be forced to stop using drugs and have time to look more introspectively at his substance use and related health (e.g., HCV) problems.

You’ll know. When you hit rock bottom you’ll know. You’ll know when you’re tired of getting high or you’ll know when you’re tired of doing whatever you’re doing. Running around, not showering, sleeping in the highways, fucking, not getting a haircut, hygiene, not, not up to par. You’ll get it … Something will click. Or even like me personally. A person like me I gotta go to jail. It’s just what it is. For me to stop, I gotta go to jail. I can’t go to detox, and I can’t go to a program. No. because I’m gonna leave. I have my own free will. I don’t got probation; I don’t have to pee in a cup. I don’t do none of that. For me you gotta lock me up in a cage for me to, a couple months, for me to get it through my head that ok this is not a way of living. I mean it’s not a way of living for nobody (not taken treatment)

### Participant experiences with HCV treatment

#### Treatment side effects

Most (*n* = 11/15) participants have completed treatment at least once. Of those who took DAA treatments, the majority had few to no side effects. Participants reported quick recovery times and feeling energetic.

But yah, I felt more energetic, I started to work, I got my license to drive, I did everything that I had to do and no one helped me. The treatment helped me. I don’t know what it is called, but it is a really good pill (taken treatment)

Three of the eleven participants who had received treatment experienced negative side effects (bedridden, weakness, headaches) from the DAA treatment. One participant who completed a 12-week treatment course expressed, “At first it wasn’t easy. Hepatitis C treatment knocks a person out … it puts you on bedrest … it makes you feel really down.” This participant expanded on the eventual decrease in the severity of the treatment saying, “When I slept, I rested more. It was something I could get through because it was sleeping. And that’s it. I continued [living] normally.” Another participant, who had relapsed using drugs, was re-infected with HCV and had begun on DAA plus ribavirin treatment said, “[I]...didn’t want to do anything. Nothing. I didn’t want to walk; I didn’t want to get out of bed. Nothing. It’s strong. It has to be strong” (completed a 24-week treatment course following a relapse).

#### Adherence to treatment

Most participants reported good adherence to DAA treatments due to shorter treatment plans and fewer side effects than older treatments. Current DAA treatments range from 8–24 weeks depending on fibrosis levels and previous treatment exposure, whereas interferon-based treatments typically lasted 24–48 weeks [19, 20]. Several participants who were co-infected with HIV/AIDS reported that they took their HCV medications more regularly than their HIV medications and that they believed that non-adherence to HCV medications could prove more fatal. “Me, for me, the virus [HIV], I took [the medication] when I wanted, I didn’t take it. Hepatitis C, no. For hepatitis C, I couldn’t miss any” (taken treatment). When asked why he did not take the HIV medications every day, he responded with, “I don’t know. I am used to taking it that way.”

Several other participants credited their doctors with helping them adhere to the DAA treatment. They reported that their doctors would regularly call to check adherence and would guide them through the process during follow-up visits. Participants felt reassured and were given resolve to continue with the treatment when they felt like quitting.

But, she [the clinician] called me, and when she called me, you know, I would tell her what was happening. You know that I was changing. But that was, like she told me, part of the medication. That was one of the side [effects], the strength of the pills, yes, what the pills did to a person. But to hold on, you know, that everything is, will be ok. And everything was ok. (taken treatment)

Patients report that physicians were also very clear about how the medication was to be taken, which contributed to adherence and sustained virologic response. One participant described the explanations he received as follows:

I know [my doctor] was calling me like once a week or something like that. Just to make sure I was taking my medication. Just to make sure that I didn’t miss a day. Just to make sure that I take it at the same time every day. You know she kept me well informed about the medication. She’d check in on me yah. (taken treatment)

#### Relapse

Several participants (*n* = 5/15) experienced drug relapses or re-contracted HCV after their initial treatment. They reported that although they initiated the treatment, they did not feel ready to start treatment and take on the responsibilities associated with this decision, such as cessation of drug use. Four of these participants have now completed a second round of treatment after drug relapse. One participant who relapsed after first completing treatment responded as follows, when asked about relapse:

I returned to the street, to drugs, because I wasn’t decided to stop the drugs. I still wasn’t sure in my mind. I wasn’t decided. (taken treatment)

One participant explained that after completing treatment or failing to do so, some people, especially Latinxs who come to Boston for treatment and have no support systems, become re-enveloped in the world of drug misuse because that is where they feel comfortable and where their friends are. Several participants reiterated this sentiment by explaining that even after they were cured of HCV, their family members and friends were always drinking and partying, and it became very difficult for them to stay sober. Alcohol often acted as a trigger for many to misuse drugs. The participant below mentions the Casa Esperanza (a local community-based agency) mantra “people, places, and things.” The sober house suggests to all its members that they should stay away from people, places, and things that may trigger their desire to use drugs.

You know people come here lost trying to make a change. But because they’re lost, they go to where they feel more comfortable. And that’s where there’s a group of addicts. And you can’t hang around the barber shop without getting a haircut. You hang around long enough, you are gonna use too. People places and things. (taken treatment)

One participant, however, was injured and was put on heavy doses of medication (prescription opioids). This medication caused him to relapse as the strong dosages triggered his need to use again.

Um, two years ago, the way I relapsed, was I had a motorcycle accident, I got run over by a truck, and I was paralyzed for 11 months, and I was hospitalized on a lot of, morphine. Percocet … And when my prescription at home, ran out, I still had the edge … I was vulnerable. I was vulnerable to, you know going back, and I knew it. And one day I was really in pain, I was really uncomfortable, I had no more meds, and I went for the quick fix you know? And I used. But that one day one using, it was a ripple effect … I ended up being picked up, I got violated by my probation. I went to jail. (taken treatment)

#### Participant suggestions on information dissemination

Participants expressed a lack of knowledge as a treatment barrier. They described that knowledge of side effects and effectiveness of new treatments were limited, and fear of these side effects often stopped many HCV-infected individuals from seeking treatment. Additionally, participants reported a lack of understanding in the Latinx community regarding steps to take along the HCV care cascade to reach treatment.

… a lot of people that I know once have mentioned the hep C they be talking about that they gonna die and this... No, no. Everything’s gonna be ok. As long as you do it the way it’s prescribed, everything’s gonna be okay. They don’t have an education and people they grow up you know I mean in the street and sometimes … you just need somebody to talk to and make them aware about what’s going on now … . (taken treatment)

Participants recommend expanding the area in which outreach and health fairs are done as they felt the information was not reaching many Latinx populations. They recommend talking to people in the streets and communities further away from the center of Boston. Methadone clinics are required by the Department of Public Health in Massachusetts to show an informative video on HCV. One participant who has been actively receiving methadone treatment for several years described her desire for a more informative educational video at the methadone clinics focusing on treatment options.

[The video] is not about the treatment. ‘Yes, you can be cured’ and things like that. If there is new information about hepatitis C, I want to hear about it because I haven’t learned anything new. Nothing that made me say, ‘wow, really?’ (not taken treatment)

## Discussion

Our study is the first to our knowledge to explore treatment readiness, experiences with, and perceptions about HCV treatment among Spanish-speaking Latinx PWID in New England, where the predominant Latinx ethnicity is Puerto Rican [21]. We found that Latinx participants reported good access to DAA therapy in Boston. They generally experienced supportive patient-provider relationships, which fostered opportunities and desires to initiate and complete therapy. However, we also found barriers to HCV treatment, as well as substance use relapse and reinfection with HCV after DAA therapy.

Access to DAAs and healthcare were widely perceived to be strong in the greater Boston area. Participants highlighted the many resources available in terms of choices of healthcare facilities, culturally competent clinicians, and translation services. Our findings differ from those of Jordan et al. and Swan et al., who found that PWID had great mistrust in their healthcare providers, felt disrespected [17], felt uncared for, and thought providers used too much medical jargon [16]. These previous studies were conducted during the era of interferon treatment, when treatment success was much lower and side effects from HCV therapies were more severe. These differences in findings are a reminder of the great variability in treatment access, as well as the policies that can facilitate and inhibit access to, initiation of, and completion of treatment [4, 13, 22].

With respect to the overall treatment experience, we found that treatment facilitators included culturally competent providers and readily available translation services. Treatment adherence was enforced when providers made regular phone calls to patients. These findings align with previous studies emphasizing that provider-patient relationships greatly improve adherence to treatment and follow-up visits [16, 17, 23].

We recruited participants who received and who did not receive HCV treatment from clinical providers. Generally, those who did not receive treatment thus far appeared to face greater barriers to treatment. In our study, treatment barriers included addiction, personal hesitations, and participant perceptions that they were not allowed to receive treatment when still injecting drugs or more than once. These findings are consistent with the current literature indicating that while there were few sobriety restrictions for MassHealth users in 2014 [13], due to physician hesitation to treat PWIDs, there was little uptake in HCV treatment among this population [24, 25]. We found, among some participants, that treatment was not sufficiently prioritized and thus not pursued, while others feared reinfection risk and the stigma and dismay that it might garner among healthcare providers and family members. Our findings are similar to those from previous research indicating that participants felt that HCV was too commonplace to cause concern, were confused about screening locations, and feared side effects from the treatment [16].

With respect to relapse, there was concern about insurance approval when going for a second HCV treatment as well as a lack of treatment readiness when those who relapsed began their initial treatment plan, findings that paralleled those in a similar study focused on English-speaking PWID in Boston [26]. Other studies have found that provider fear of drug use relapse has created barriers to treatment access [24, 27, 28].

Among participants in our study who experienced a drug injecting relapse, two reasons were provided: (1) they had not felt entirely ready to stop using drugs prior to HCV treatment initiation, and after initial sustained virologic response^1^ was achieved [2], social circles that promoted alcohol and substance misuse triggered participants to relapse. Other studies have highlighted the importance of breaking ties with drug-using associates [29], and the need for strong social support to help cope with cessation of drug use [30, 31].

Our study has several limitations. First, all but one participant was Puerto Rican, which limited our understanding of the wider Latinx population’s sentiments. However, the largest proportion of Latinxs in Boston is Puerto Rican, so our study provides critical information about the perceptions of primarily Spanish-speaking Puerto Ricans [21]. Second, our study has a relatively high percentage of participants who are already connected to care. Most participants had already received HCV treatment and had similar treatment experiences, and their experiences were different from those of participants we recruited through street outreach. Still, our study provides initial insights into the successes, challenges, and lessons learned relevant to HCV treatment perceptions and experiences among Latinx PWID. Finally, because HCV is a larger burden among males who inject drugs [8], our study primarily includes men. However, these demographics limit insights into experiences and perspectives among women who inject drugs.

## Conclusions

Our study fills important gaps in knowledge of and experiences with HCV treatment among Spanish-speaking Latinxs. Most Latinx PWID in our sample reported positive experiences overall with HCV treatment during the DAA era, and many highlighted the strong relationships with clinical providers that helped assure HCV treatment and adherence. Barriers to treatment included misinformation about insurance options, fear of side effects, and ongoing drug use. Primary reasons for HCV re-infection included lack of readiness when initiating treatment or continued injection drug use while on treatment. Lack of social support and frequent proximity to substance use triggers were also cited as strong influences for risk of HCV infection. Participants reported a lack of knowledge about HCV in Boston communities and recommended widening the perimeter of outreach programs to ensure that information on differences between new and old HCV treatments could be highlighted. They also recommended a step-by-step plan to ensure that one reaches the end of the treatment cascade.

## Data Availability

Data may be made available upon written request to the senior author and PI on the study (Stopka)

## Abbreviations

AIDS: Acquired immunodeficiency syndrome
DAA: Direct-acting antivirals
HCV: Hepatitis C virus
HIV: Human immunodeficiency virus
PR: Puerto Rican
PWID: People who inject drugs

## Footnotes

1 Sustained virologic response: the patient has an undetectable HCV RNA level 24 weeks after treatment was completed.
